# Brainstem metastases treated with Gamma Knife stereotactic radiosurgery: the Indiana University Health experience

**DOI:** 10.2217/cns-2017-0029

**Published:** 2017-12-14

**Authors:** Ajay Patel, Homan Mohammadi, Tuo Dong, Kevin Ren-Yeh Shiue, Douglas Frye, Yi Le, Shaheryar Ansari, Gordon A Watson, James C Miller, Tim Lautenschlaeger

**Affiliations:** 1 Indiana University School of Medicine, Indianapolis, IN 46202, USA; 2 Department of Radiation Oncology, Indiana University School of Medicine, Indianapolis, IN 46202, USA; 3 Goodman Campbell Brain & Spine & Department of Neurological Surgery, Indiana University, Indianapolis, IN 46202, USA

**Keywords:** brainstem, Gamma Knife, metastasis, radiosurgery

## Abstract

Brainstem metastases offer a unique challenge in cancer treatment, yet stereotactic radiosurgery (SRS) has proven to be an effective modality in treating these tumors. This report discusses the clinical outcomes of patients with brainstem metastases treated at Indiana University with Gamma Knife (GK) radiosurgery from 2008 to 2016. 19 brainstem metastases from 14 patients who had follow-up brain imaging were identified. Median tumor volume was 0.04 cc (range: 0.01–2.0 cc). Median prescribed dose was 17.5 Gy to the 50% isodose line (range: 14–22 Gy). Median survival after GK SRS treatment to brainstem lesion was 17.2 months (range: 2.8–45.6 months). The experience at Indiana University confirms the safety and efficacy of range of GK SRS prescription doses (14–22 Gy) to brainstem metastases.

**Summary points**

- Stereotactic radiosurgery is an effective and well-tolerated treatment for brainstem metastases.
- The risk of grade 3 or greater toxicity from radiation is around 3% in the literature.
- Local control at 12 months is 74–100% in the existing literature.
- This data at one institution compare favorably to the existing data in the literature.
- The overall prognosis for brainstem metastases remains poor.
- Due to the rare incidence of brainstem metastases, analysis of variables impacting outcomes remains difficult.
- Relatively higher doses appear safe and might be worth investigating further.
- Further investigations, with respect to personalized radiation dosing accounting for histology and other factors might improve cancer control and toxicity profile.

The brain is a very common site of metastasis for malignant tumors, affecting 9–17% of all cancer patients at some point in their disease course [1]. Approximately, 80% of brain metastases occur in the cerebrum, another 10–15% in the cerebellum and about 2–3% in the brainstem [2]. Although brainstem metastases are rare, they are associated with the worst prognosis with a survival rate of 1–6 months without directed therapy [3,4]. Brainstem metastases (BSM) are often inoperable, compromised by the density of functional fiber tracts converging in a small cross sectional area/volume. In the past few decades, stereotactic radiosurgery (SRS) has proven to be an effective modality in the treatment of these tumors. SRS offers excellent local control of tumors ranging between 74–100% and a median reported survival after SRS of 4–12 months [3–29]. Although effective in local control, due to the high dose to fraction ratio, toxicity is a critical concern. SRS for brainstem metastases has been reported to be associated with severe or life-threatening toxicity in 0–9.5% of patients [3–29]. This report reviews Indiana University's (IU) experience with Gamma Knife (GK)-based SRS for brainstem metastases with respect to local control, overall survival (OS) and toxicity within the brainstem based on lesion size and radiation dose.

## Methods

### Patient population

This study is an Institutional Review Board-approved single institution retrospective review. Patients were identified via medical billing codes and chart review through the hospital-based medical record system (Cerner) as well as the radiation record and verification system (ARIA, Varian Medical System). Patients from 2008 to 2016 with secondary neoplasms to the brain treated with GK SRS were identified. Patients, 18 years or older with a definitive diagnosis of a primary tumor who were noted to have secondary metastasis to the brainstem found on MRI or computed tomography were included in the study. Locations of the brainstem were categorized as: midbrain, pons or medulla. The presence of other brain metastases (including those treated with any modality) was not an exclusion criteria. Patients that had no follow-up after their GK SRS treatment were excluded.

All patients received preoperative neuroimaging with MRI. All end points were assessed starting from the date of GK SRS treatment, unless stated otherwise. Follow-up time was determined by the date of either last imaging or last visit with a cancer specialist (medical oncologist, radiation oncologist or neurosurgeon). OS was determined by confirming the date of death of the patient and censored to the last imaging or clinical follow-up, whichever occurred later in time. The distant brain failure-free survival after GK SRS treatment to brainstem lesions was determined by the first date of MRI demonstrating growth of other intracranial metastases and censored to last clinical follow-up, last imaging follow-up or death; which ever occurred later in time.

The data analyzed for SRS included: OS from brainstem SRS date, prescribed dose, prescription isodose level, maximum dose to gross tumor volume (GTV), minimum dose to GTV, brainstem metastasis diameter, brainstem metastasis volume, time to local failure and time to distant brain metastases failure. Change in tumor size was also investigated by examining image reads already in the records, with priority given to the definitive radiology interpretation. Treatment-related toxicity was assessed by examining clinical notes to determine any new neurological symptoms and by reviewing neuroradiology reads. In order to better detect the presence of toxicity we also reviewed clinical records to see if patients were started on corticosteroids post-GK SRS and whether the brainstem lesion was the culprit.

### Radiosurgery technique

A Leksell model G stereotactic frame was attached to the patient's head via four pin sites by a neurosurgeon and MRI with gadolinium contrast was obtained with the head frame in place. The MRI was loaded into the GammaPlan system and the tumor was then outlined. Treatment planning focused on maximizing coverage and conformality. The plan was approved by a neurosurgeon, a radiation oncologist and the medical physics team. Prescription doses varied over time due to different treating GK physicians over the last decade.

### Statistical analysis

We identified 14 patients with 19 brainstem metastatic lesions treated with GK SRS. Data were analyzed via the Kaplan–Meier method. Kaplan–Meier plots were generated for OS, local control and distant brain failure-free survival; all after treatment of the brainstem lesions with GK SRS. The data were censored to either the last oncologic follow-up or last imaging follow-up, which ever occurred later in time. For local control and distant brain failure-free survival, data were also censored to death.

## Results

### Clinical results

From 2008 to 2016, 14 patients who had 19 brainstem metastases met eligibility criteria for inclusion in this study. Imaging follow-up varied from 1.2 to 43.6 months (median: 15.3 months). Oncologic follow-up varied from 1.6 to 44.5 months (median: 10.0 months). Table 1 lists patient, tumor and GK SRS characteristics. Age at treatment ranged from 37.3 to 70.9 years old (median: 56.3 years old). The male to female ratio was 1:1.3. Karnofsky Performance Status score for patients preradiosurgery ranged from 70 to 90 (median: 85). Two patients did not have a Karnofsky Performance Status score in their records. The most common primary tumor was non-small-cell lung carcinoma (35.7% of patients). The most common site for brainstem metastases was the pons (68.4% of lesions). The most common lesion laterality was right (63.1% of lesions). A single lesion was treated in 71.4% of patients, two lesions in 21.4% of patients and three lesions in 7.1% of patients. Active extracranial disease was present in 78.6% of patients. At the time of GK SRS, 28.6% of patients had a single additional intracranial lesion and 71.4% had two or more. Whole-brain radiation therapy (WBRT) was performed in 35.7% of patients’ preradiosurgery and 7.1% of patients postradiosurgery.

**Table 1.** **Clinical characteristics of 19 brainstem metastatic tumors in 14 patients.**

**Characteristic**	**Value or median**	**Percentage or range**
Number of patients	14	

Brainstem lesions treated	19	

Age of diagnosis of brainstem met	56	37–71

Age at treatment (years)	56	37–71

**Sex**		

– Male	6	42.90%

– Female	8	57.10%

**KPS (12 patients)**		

– Median KPS	85	70-90

**Brainstem lesion location**		

– Midbrain	3	15.80%

– Pons	13	68.40%

– Medulla	3	15.80%

**Number of brainstem metastases treated per patient**		

– 1	10	71.40%

– 2	3	21.40%

– 3	1	7.10%

– >3	0	

**Total intracranial metastases present per patient**		

– 1	4	28.60%

– 2	3	21.40%

– 3	0	0.00%

– 4	0	0.00%

– 5	2	14.30%

– >5	5	35.70%

**Brainstem lesion laterality**		

– Right	12	63.20%

– Left	5	26.30%

– Other	2	10.50%

**Prior WBRT in patients**		

– Yes	5	35.70%

– No	8	57.10%

– After	1	7.10%

**Primary histology**		

– NSCLC	5	35.70%

– SCLC	1	7.10%

– Breast carcinoma	2	14.30%

– RCC	3	21.40%

– Melanoma	2	14.30%

– Apocrine carcinoma of L. axilla	1	7.10%

Median time from primary to brainstem diagnosis (months)	39.2	(-1.0)–(101)

Follow-up duration (months)	10	1.6-44.5

Imaging follow-up (months)	15.3	1.2-43.6

**Extracranial metastases present at time of SRS for patients**		

– Yes	11	78.60%

– No	3	21.40%

**Primary controlled at time of brainstem lesion treatment**		

– Yes	9	64.30%

– No	5	35.70%

**SRS**		

**OS (months)**		

– Median from first brainstem SRS date	17.2	2.8–45.6

Prescribed dose (Gy)	17.5	14–22

Isodose level (%)	50	50–70

Maximum dose (Gy)	33	25.8–39.3

Minimum dose to GTV (Gy)	18	13.8–30.6

Brainstem metastasis diameter (cm)	0.6	0.2–1.7

Brainstem metastasis volume (cc)	0.04	0.007–2.0

**Local control of lesion (months)**		

– 6	17	100%

– 9	15	93.80%

– 12	8	87.50%

Time to distant brain failure (months)	8.4	1.2–45.6

GTV: Gross tumor volume; Gy: Gray; KPS: Karnofsky Performance Status; NSCLC: Non-small-cell lung carcinoma; L. axilla: Left axilla; OS: Overall survival; RCC: Renal cell carcinoma; SCLC: Small-cell lung carcinoma; SRS: Stereotactic radiosurgery; WBRT: Whole-brain radiation therapy.

### SRS results

For the 19 lesions brainstem tumor size varied from 0.01 to 2.0 cc (median: 0.04 cc). The prescribed dose varied from 14 to 22 Gy (median: 17.5 Gy). The isodose level varied from 50 to 70% (median: 50%). The maximum dose delivered to GTV varied from 25.8 to 39.3 Gy (median: 33 Gy). The minimum dose delivered to GTV varied from 13.8 to 30.6 Gy (median: 18 Gy).

### Local control, survival & toxicity

**Figure 1.**
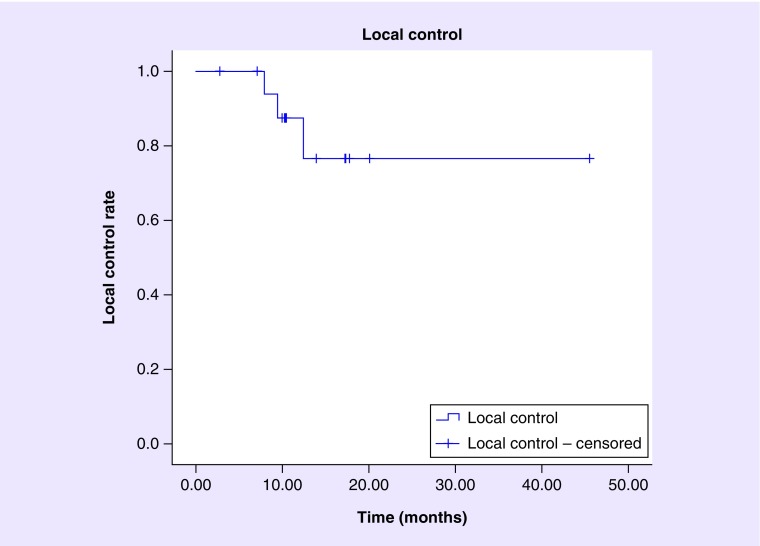
**Kaplan–Meier analysis of local control after Gamma Knife stereotactic radiosurgery treatment of brainstem metastases.** Local control rate at 6, 9 and 12 months was 100, 93.8 and 87.5%, respectively, The number at risk at 0, 6, 9 and 12 months was 19, 17, 15 and 8, respectively. Data were censored to last clinical follow-up, last imaging follow-up or death, whichever occurred later.

Local control rates at 6, 9 and 12 months were 100, 93.8 and 87.5%, respectively, with the number of lesions at risk of failure at 0, 6, 9 and 12 months being 19, 17, 15 and 8, respectively. Figure 1 demonstrates the Kaplan–Meier plot with respect to local control for the 19 lesions.

**Figure 2.**
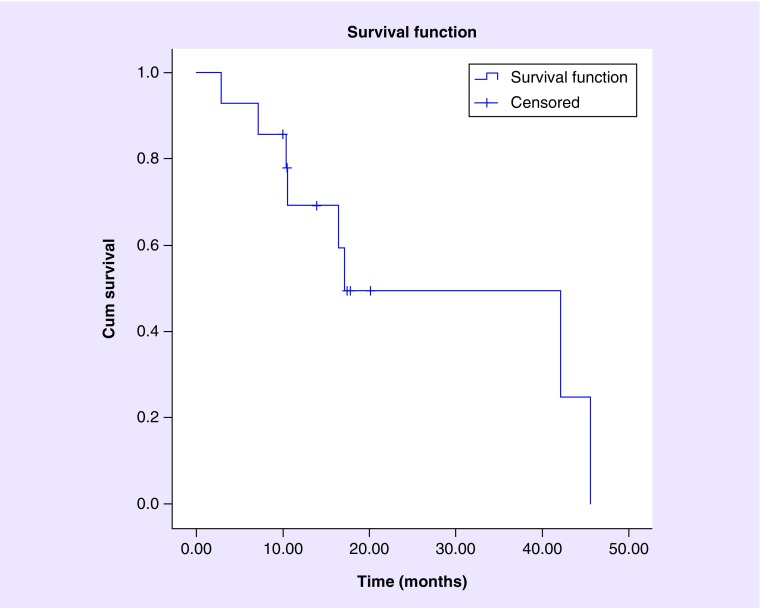
**Kaplan–Meier analysis of overall survival after Gamma Knife stereotactic radiosurgery treatment of brainstem metastases.** Median overall survival was found to be 17.2 months. Overall survival rate at 6, 9 and 12 months was 92.9, 85.7 and 69.3%, respectively. The number at risk at 0, 6, 9 and 12 months was 14, 13, 12 and 8, respectively. Data were censored to last clinical follow-up or last imaging follow-up, whichever occurred later.

Median OS was found to be 17.2 months. OS rates at 6, 9 and 12 months were 92.9, 85.7 and 69.3%, respectively. With the number of patients at risk of death at 0, 6, 9 and 12 months being 14, 13, 12 and 8, respectively. Figure 2 demonstrates the Kaplan–Meier plot with respect to OS of patients in this study.

**Figure 3.**
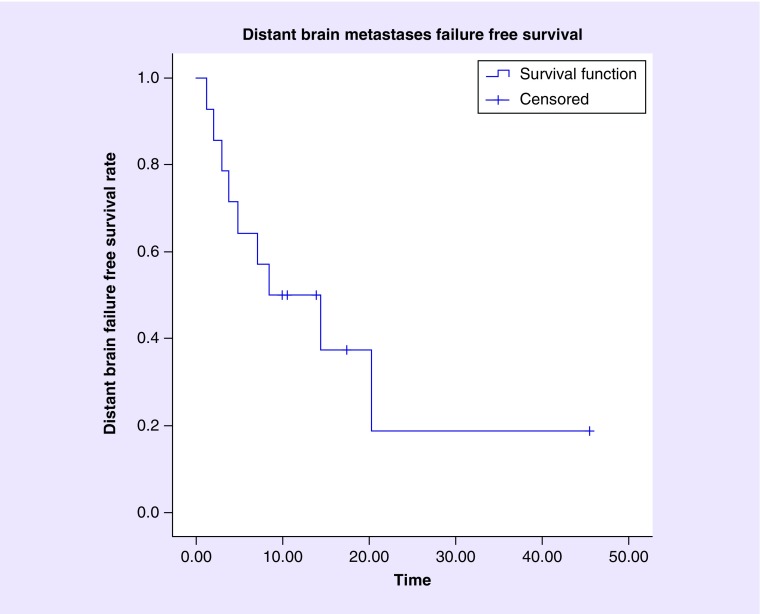
**Kaplan–Meier analysis of distant brain failure-free survival after Gamma Knife stereotactic radiosurgery treatment of brainstem metastases.** The median time to distant brain failure was 8.4 months. Distant brain failure rate at 6, 9 and 12 months was 64.3, 50 and 50%, respectively. The number at risk at 0, 6, 9 and 12 months was 14, 9, 7 and 5, respectively. Data were censored to last clinical follow-up, last imaging follow-up or death, whichever occurred later.

The median time to distant brain failure was 8.4 months. Distant brain failure rate at 6, 9 and 12 months was 64.3, 50 and 50%, respectively, with the number of patients at risk of distant brain failure at 0, 6, 9 and 12 months being 14, 9, 7 and 5, respectively. Figure 3 illustrates the Kaplan–Meier plot of distant brain failure-free survival after treating brainstem metastatic lesions via GK SRS.

There was no observed grade 3 or greater toxicity at our institution with respect to brainstem metastases after treatment with GK radiosurgery [30].

## Discussion

GK SRS has consistently been shown to be an effective modality in the treatment of brainstem metastases [3–29,31–32]. This report describes the IU experience with GK brainstem radiosurgery over a 9-year time frame. In the study, there was a median prescribed dose and a median maximum dose of 17.5 and 33 Gy, respectively. In prior literature, median margin doses ranging from 13 to 18 Gy have been published. Local control rates of 100% at 6 months and 87.5% at 12 months were observed, which is consistent with that reported in prior literature ranging from 74 to 96% at 12 months [3–29,31–32]. In particular, the International Brainstem SRS Study by Trifiletti *et al*. reported a local control rate of 81.8% at 1 year [27]. No toxicity of any grade was observed, which is consistent with the 0–9.5% incidence of severe or life-threatening toxicity in prior literature, in other words grade 3 or greater [3–29,31–32]. In this study, improved local control compared with previous studies was noticed, especially those that were treated with lower marginal doses. There are only five manuscripts that report a median marginal dose greater than 17 Gy [3–29,31–32]. The IU study and existing data suggest that marginal doses greater than 17 Gy are safe and possibly associated with improved local control, while still allowing for minimal toxicity.

Two cases are discussed more particularly. The first, a 58-year-old female with a well-controlled breast adenocarcinoma, who also had a right pontine lesion adjacent to the foramen of Luschka. The primary was ER/PR+ and Her2-. The patient did not receive WBRT. The patient had a total number of two new brain lesions. The brainstem metastatic tumor volume was 0.2 cc. The prescribed dose was 20 Gy to the 51% isodose line. Last imaging follow-up was 10.5 months from the date of treatment and at that time there was no evidence of lesion failure, radionecrosis or hemorrhage at the treated site. The second case was a 55-year-old male with clear cell renal cell carcinoma and ten intracranial metastases; two of which were brainstem lesions. The first brainstem lesion was a left medulla lesion of volume 0.02 cc treated with a prescribed dose of 17 Gy with no toxicity or local failure at the last imaging follow-up at 20.0 months. The second brainstem lesion was a left pontine lesion of volume 0.04 cc treated with a prescribed dose of 22 Gy to the 60% isodose line with a maximum dose to GTV of 36.8 Gy and minimum dose to GTV of 24 Gy. The second lesion also did not demonstrate failure or develop toxicity at last imaging follow-up at 7.9 months. The two treated brainstem lesions remained stable. These two cases demonstrate that when treating brainstem metastases with GK SRS, higher doses may be safer than previously thought. This finding warrants further investigation.

Previous publications report median margin dose ranges between 13 and 18 Gy. WBRT prior to or after SRS ranges from 6.5 to 96.4% with the mean being 49.8 ± 20.0%. The local control rate at 12 months varies from 74 to 100%. The median OS ranges from 3.9 to 12.0 months. The local control rate at 12 months based on the mean of all the reported values in literature turns out to be 86.5 ± 6.2%. Rate of toxicity reported in patients treated with SRS for BSM varied from 0 to 9.5%. The average rate of toxicity based on reported percentages per report was 3.8 ± 3.5% [3–29,31–32].

An important factor to note is the brain tumor volumes in the IU study were relatively small ranging from 0.01 to 2.0 cc with a median of 0.04 cc, while the median target volumes reported in literature range from 0.1 to 2.8 cc [3–29,31–32]. The increased local control observed in our cohort, along with the low incidence of toxicity, is likely attributable to the relatively higher dose being delivered to a smaller volume. While there was heterogeneity in terms of extracranial and intracranial disease state, which certainly affects OS; the OS and follow-up times in this manuscript allows reasonable reporting of toxicity and local control.

This study has all the limitations of a standard retrospective database review including but not limited to a heterogeneous patient population with confounding variables, including selection bias and patient comorbidities. Most notably, selection biases are not well controlled in this setting. Additionally, our survival data only extend to the last clinical follow-up or last MRI follow-up, while in reality these patients may have survived longer. The low incidence of toxicities may be due to a lead time bias as some of these patients have passed away from primary tumor burden or were lost to follow-up before any toxicity could develop or be observed. The investigation also had a relatively smaller sample size as compared with prior studies. Further prospective investigations into the benefits and risks of dose intensification are warranted.

## Conclusion

GK SRS prescription dose ranges of 14–22 Gy are safe and effective in the treatment of brainstem metastases. At these doses, no significant toxicity was observed. Rather, favorable local control and OS was found compared with prior published studies. Treatment of smaller lesions with higher prescription doses was effective despite their anatomically unfavorable location. This study indicates the potential value in prospectively evaluating local control and toxicity with higher marginal doses, particularly in smaller lesions, than previously thought safe for brainstem metastases treated with GK SRS.
